# Risks, benefits and survival strategies-views from female sex workers in Savannakhet, Laos

**DOI:** 10.1186/1471-2458-12-1004

**Published:** 2012-11-20

**Authors:** Ketkesone Phrasisombath, Elisabeth Faxelid, Vanphanom Sychareun, Sarah Thomsen

**Affiliations:** 1 Faculty of Postgraduate Studies and Research, University of Health Sciences, Vientiane, Laos; 2 Department of Public Health Sciences, Division of Global Health (IHCAR), Karolinska Institutet, Stockholm, SE 171 77, Sweden

**Keywords:** Risk, Benefit, Female sex worker, Savannakhet, Laos

## Abstract

**Background:**

Female sex workers (FSWs) are vulnerable to sexually transmitted infections (STIs) and encounter socio-economic and health problems, including STIs/HIV, unintended pregnancy and complications from unsafe abortion, stigma, violence, and drug addiction. Reducing risks associated with sex work requires an understanding of the social and cultural context in which sex workers live and work. This study aimed to explore the working environment and perceived risks among FSWs in Savannakhet province in Laos.

**Methods:**

Five focus group discussions (FGDs) and seven interviews were conducted with FSWs in Kaysone Phomvihan district in Laos. Latent content analysis was used to analyze the transcribed text.

**Results:**

The results revealed that the FSWs were aware of risks but they also talked about benefits related to their work. The risks were grouped into six categories: STIs/HIV, unintended pregnancy, stigma, violence, being cheated, and social and economic insecurity. The reported benefits were financial security, fulfilling social obligations, and sexual pleasure. The FSWs reported using a number of strategies to reduce risks and increase benefits.

**Conclusions:**

The desire to be self-sufficient and earn as much money as possible put the FSWs in disadvantaged and vulnerable situations. Fear of financial insecurity, obligations to support one’s family and the need to secure the future influenced FSWs’ decisions to have safe or unsafe sex. The FSWs were, however, not only victims. They also had some control over their lives and working environment, with most viewing their work as an easy and good way of earning money.

## Background

Female sex workers (FSWs) are at risk of heterosexual transmission of sexually transmitted infections (STIs) including human immunodeficiency virus (HIV) because of inconsistent condom use, multiple sexual partners, and sexual violence from their sexual partners [1-3]. In Laos, the main route of STI and HIV transmission is through sexual contact with an infected partner [4]. In 2005, the Government of Laos approved a National Strategic and Action Plan on STI/HIV for 2006–2010. The aim was to expand national capacity in achieving universal and non-discriminating access to treatment, and care and support in order to improve the HIV situation throughout the country. The target groups included female sex workers (FSWs), migrant workers, men who have sex with men, and drug users [5]. Despite these efforts, between 0.8 percent and 4.2 percent of FSWs are estimated to be infected with HIV [6], and the HIV prevalence among men who have sex with men is 5.6 percent [7].

Commercial sex, although a visible phenomenon in Laos, is illegal. Due to perceived inappropriate behavior according to the Lao traditional culture, FSWs were previously put in rehabilitation camps called *Don Nang* or ‘women′s island’ [8]. Although this is no longer practiced, studies have shown that FSWs in Laos are still perceived negatively and stigmatized, especially by healthcare providers (HCPs) [9]. Furthermore, seeking testing and treatment for STIs is hindered by high costs, long waiting times, and judgmental attitudes of HCPs [10]. FSWs are also vulnerable to sexual and reproductive illness including STIs and unsafe abortion [1], not only because of their risky sexual behaviour but also because of economic insecurity and obligations to support the family (Phrasisombath K, Thomsen S, Sychareun V, Faxelid E: ‘Health is Wealth and Wealth is health’-perception of health and ill-health among female sex workers in Savannakhet province, Laos, 2011. (Under review, Global Health Action, 2012)).

In Laos, efforts to develop effective interventions to reduce STIs and HIV transmission among FSWs have remained limited. One possible explanation is that most approaches to prevention have focused on individual activities but have not considered the reality of FSWs e.g. decision-making in relation to safe sex. Reducing risks associated with sex work in Laos requires an understanding of the social and cultural context in which FSWs live and work. Previous studies have mainly focused on condom use, number and types of sexual partners [6], knowledge about STIs/HIV, and prevalence data on STI and HIV [11]. Issues related to FSWs’ vulnerability and risks related to their working situation are poorly understood. In this study, we explore the working environment and perceived risks and benefits among FSWs in Savannakhet province in Laos.

## Methods

### Setting

The study was based in Savannakhet province, which has the highest rate of STI and HIV-infection reported among FSWs in Laos [4]. Savannakhet comprises 15 districts with one provincial and 14 district hospitals. The province has approximately 826,000 inhabitants, of which women represent 51 percent. About 77 percent of the population live in rural areas [12]. The main sources of income in the province are from gold mining, cement production, rubber plants, and sugar production [13]. In 2003, care and support for people with HIV/AIDS was initiated in a pilot project providing antiretroviral treatment (ART) in Kaysone Phomvihan, the main district of the province. In addition, a programme called ‘100% condom use program’ was implemented [4]. The bar owners or *mamasans* (pimps) in the target districts are involved in providing health information and supporting FSWs to have regular check-ups for STI and to visit health facilities when ill. In 2006, a drop-in centre was established in Kaysone Phomvihan. The centre is under supervision by the Secretariat of HIV/AIDS/STI Prevention and Control Unit, Savannakhet Province (PCCA). Activities are designed for high-risk groups such as FSWs, blood donors, migrant workers and those requesting anonymous tests. In 2008, a voluntary counseling and testing (VCT) program for HIV was implemented and about 3,000 individuals have been tested so far. The majority of those HIV positive are FSWs and migrant workers. The counselling and testing is implemented in a confidential manner and available to patients who are in need. Behaviour communication change (BCC), condom distribution, advocacy for condom use, and treatment of reproductive tract infections (RTI) and STIs are free of charge and in 2008 those activities reached about two-thirds of the estimated 450 FSWs in the province [14]. However, if medicines are limited or not available the patients will get a prescription and be requested to buy the recommended medicines in a pharmacy using their own money. Recently, the drop-in centre and the 100% condom use program have extended their services and now collaborate with about two-thirds of the district hospitals in the province. Kaysone Phomvihan district was selected as the study site because there are many entertainment places where FSWs work and live in this district.

There are two types of FSWs in Laos, street-based FSWs and venue-based FSWs, although there are no street-based FSWs in Savannakhet province. Venue-based FSWs are women who are officially employed as hostesses to work in entertainment places (e.g. beer bars ‘drinkshops’, karaoke bars, nightclubs, guesthouses and restaurants) to provide services to clients in the form of serving beer, snacks and conversation but also selling sex [5]. Venue-based FSWs find clients through pimps and provide sexual services in guesthouses or hotels or in the client’s room, which are usually attached to the entertainment places. Sex workers may entertain the clients for a shorter or longer period. If the woman wants to join the client after the bar has closed, they can also go together to other settings.

### Design and research team

Qualitative data collection methods were employed. Focus group discussions (FGDs) explored the views of the group, whereas in-depth interviews explored individuals’ views and experiences [15]. The research team consisted of two Swedish researchers, (ST) a social scientist and (EF) a midwife, both specialists in international public health research, specifically sexual and reproductive health aspects, two Lao researchers (KP, VS) both with a medical specialty, one peer educator from the drop-in centre who previously used to work as sex worker and who had daily contact with FSWs to give condoms and provide health information, and finally one female interviewer with a background in social sciences. Before the study started, the research team conducted a mapping procedure to identify entertainment establishments and FSW in the study area. This was done in a previous study conducted by the authors from January to March, 2010. In total, 194 FSWs and 72 bars in Kaysone Phomvihan were identified [16]. Although we had previously conducted a mapping in the area, the number of FSWs may change since the women often change location and some entertainment places may have closed during the study. Therefore, we obtained information about FSWs and number of entertainment places from the drop-in centre as the centre has regular contact with FSWs using peer educators and has monthly records of numbers of SWs and bars. Furthermore, we compared the information from the centre to data from our mapping which was similar. Members of the Lao research team contacted FSWs in Kaysone Phomvihan before the data collection started. During this initiation period, the Lao research team members visited and talked to the FSWs several times in order to create rapport and build trust.

### Procedure and tool

A three-day training course on how to perform qualitative interviews and moderate group discussion was conducted for the research assistants. This training was lead by EF and the first author. The interview guide had questions related to general background of the informants, their views about the working situation and connected risks, and how the FSWs coped with their situation. The FGD guide included questions about the women’s views about their work, risks related to their work, perceptions about how other people look upon FSWs, how to be accepted by the community, and how and where to find support. The first author and the interviewer conducted one in-depth interview and one FGD each in order to test the interview questionnaire, the FGD guide, and the procedures for sampling and data collection (e.g. if a male moderator/interviewer was accepted by the participants). The results of these pilot interviews and discussions were shared and discussed with the rest of the research team. Minor modifications of the guide were made in order to ensure that the guide was suitable, culturally acceptable, and the words used not too sensitive. The participants talked freely and seemed to accept a male interviewer/moderator. We observed no differences in information obtained from FGDs and individual interviews between male and female interviewers/moderators. The interactions in the FGDs were also open and free. The results from these pilot interviews and FGDs are included in the analysis.

### Participants and data collection

Women who stated that they were presently working as sex workers and who were willing to be interviewed and participate in a FGD were recruited. The women were purposively selected from different entertainment establishments based on age, marital status, duration of sex work, and type of workplace. The FGDs and the individual interviews were carried out during March to April 2010 in a private room arranged by the research team. The first author moderated two FGDs and the female interviewer moderated three. The number of participants in the FGDs ranged between 7 and 9 [17]. Women who provided rich information during group discussions, e.g. “key informants” (KIs), were asked to come back for an interview the following day. The first author conducted three interviews and the female interviewer conducted four. Both FGDs and interviews were carried out in Laotian. The data collection was stopped when no new information from FGDs and KIs could be retrieved; in order words, at the point of saturation. In all, five FGDs with 39 women and seven interviews were performed. Interviews and FGDs lasted between 45 and 90 minutes each and were taped-recorded with the participants’ permission.

### Data analysis

The tape-recorded group discussions and interviews were transcribed into Laotian by the female interviewer and the first author. Two interviews and one FGD were translated into English and shared with the English-speaking research team members in order to understand the data and provide comments during the analysis. The transcripts were analyzed using latent content analysis, which is a stepwise analytical process, focusing on description and interpretation of underlying meanings of the text [18]. The first author initiated the analysis by reading the transcripts several times in order to obtain overall meaning of the text. Through an inductive process, meaning units were identified. Data were condensed afterwards and labeled with a code. One of the supervisors (ST) read and coded the interviews and FGD that were translated to English separately. The codes were compared and discussed until we reached consensus. Codes that reflected the core meanings in the text were merged into categories. Two themes, perceived risks and strategies to reduce risks, and perceived benefits and strategies to increase benefits emerged. In order to reduce misinterpretations the transcriptions and the taped-recorded interviews were used side-by-side throughout analysis. The research team discussed the procedures and the findings until they agreed on categories and themes.

### Ethical considerations

The National Ethics Committee for Health Research, Laos, the Regional Ethics Committee in Stockholm, Sweden, and the local authority in Savannakhet province approved the informed consent procedures of the study.

## Results

The sex workers told us about their working situations, including their perceived risks. But they also talked about the benefits of sex work, as well as the strategies that they used to a) reduce risks and b) increase benefits. Below, we describe this holistic view of the working environment of sex workers in Savannakhet province from their own perspective.

### Perceived risks and strategies to reduce risks

Participants reported six categories of risks related to their work: 1) STIs/HIV, 2) unintended pregnancy, 3) violence, 4) stigma, 5) being cheated, and 6) social and economic insecurity. For each of these risks, participants had a set of strategies for reducing them (Table 1). These risks and related strategies are presented below.

1) Risks of STIs/HIV and strategies to reduce this risk

**Table 1:** Negative outcomes for FSWs in Laos and strategies to avoid them

**Strategies**	**Risks**
Deny sex without a condom	STIs/HIV
Don’t use the client’s condom	
Carry condoms	
Refuse oral or anal sex	
Refuse sex without a condom	
Discuss type of sexual services	
Instruct client to use a condom	
Use condoms	Pregnancy
Use contraceptives	
Use condoms and birth control medicines	
Refuse sex without a condom	
Apply a withdrawal technique	
Observe and select client	Violence
Escape impolite client	
Bring mobile phone	
Reject drunk “harsh” client	
Pay police bribes	
Do what client wants	
Be nice to client	
Hide job and working status	Stigma
Escape when risk of being disclosed	
Avoid social contact	
Have abortion to keep social image	
Financially support the family	
Need to be healthy when go home	
Wear expensive clothes and dress properly	
Participate in village activities	
Observe and select client	Being cheated
Carry small amounts of money	
Ignore impolite “harsh” clients	
Reject drunk clients	
Discuss type of sex act and price	
Ask for money in advance	
Inform friend of whereabouts	
Get information from friends	
Pay police bribes	Social and economic insecurity
Serve the needs of clients	
Pretend to do what client wants	
Avoiding having a boyfriend	
Abortion to keep work	
Hide STI symptoms	
Don’t fight the client	

Risks of STIs/HIV were extensively reported in all FGDs and in-depth interviews. Discussions indicated that sex without a condom was perceived as a source of acquiring STIs/HIV. However, alternative sex acts (oral and/or anal sex) were also perceived to be risky. The most common source of risk that sex workers expressed was when clients took off a condom during the sexual act or if the condom broke: “Clients use a condom when starting intercourse but later take it off in between. This is very bad. This may lead us to die because of AIDS” [KI, aged 18 from nightclub]. Some women reported that they did not accept alternative sex acts requested by their clients because of the risk involved. Many perceived that oral sex was “dirty” because the mouth is for “eating rice”.

"“With oral sex you risk swallowing STIs/HIV into the stomach while sucking the cock, whereas anal sex is painful and later causes infections. Both are risks, so there is no need to do these” [KI, aged 23 from beer bar & restaurant]."

Sex workers had different strategies for reducing risk. In all five FGDs the participants said that using condoms is an effective preventive measure to prevent STIs/HIV.

"“Our work is risk, we use a condom to protect ourselves, if we don’t use a condom’ we would get infected and die because of AIDS” [17–31 years old FSW in FGD3]."

Condom breakage or clients removing the condom during sex was difficult to prevent. To manage this, a woman carried her own condoms that she knew were reliable:

"“You will never know how many condoms the clients will use and at night it is difficult to find even one condom. The best way is to carry many good condoms” [KI, aged 26 from beer bar]."

Women learned through experience how to avoid making mistakes with condoms, such as observing if clients used reliable condoms, checking the expiration date, and double-checking that the condom was still in place:

2) Unintended pregnancy and strategies to reduce risk of pregnancy

"“We look at the condom when the clients change sexual position or use our fingers to touch the cock in between the transaction to feel if the condom is still in place or not” [21–31 years old FSW in FGD5]."

The second source of risk that featured clearly in sex workers′ work environment was unintended pregnancy. Many women reported getting pregnant with an unknown client after engaging in sex work: “I got pregnant after I was at this work for three months” [KI, aged 18 from nightclub]. Some women said pregnancy is not easy to avoid: “Sex workers are at risk of pregnancy when clients force to have sex without a condom” [17–26 years old FSW in FGD4].

Another woman explained that pregnancy out of wedlock also meant a risk of social stigma and great shame:

"“If we go home pregnant, our parents would get seriously angry and complain like ‘I have a bad daughter’… They would not listen to us because they would feel ashamed that their daughter is pregnant without Basy ‘wedding ceremony’” [18–31 years old FSW in FGD3]."

Sex workers’ primary strategy to avoid pregnancy was to use another contraceptive method in addition to a condom (e.g. dual protection).

"“Condoms can prevent STIs/HIV, but not a pregnancy. If I use both condoms and oral contraceptive pills it is safer. This way, when the condom is ruptured the pill still protects me from an unwanted pregnancy” [KI, aged 24 from beer bar & nightclub]."

Another woman commented that when having sex with a boyfriend it is not possible to use a condom, thus increasing the risk of pregnancy. Therefore she used the withdrawal method:

"“When I have sex with my boyfriend we do not use a condom but we use the withdrawal technique to avoid risk of pregnancy” [KI, aged 23 from beer bar & guesthouse]."

Some women said that they did not apply the “withdrawal technique” with clients because of fears of getting STIs or pregnancy. One strategy for avoiding being a social outcast due to pregnancy was to have an abortion. This is illustrated below:

3) Violence and strategies to reduce risk of violence

"“If my parents knew that I got pregnant then I would absolutely be killed. No matter how much money someone paid me to keep the baby, I would have an abortion” [KI, aged 19 from beer bar & karaoke bar]."

The third major source of risk was violence from clients. This included non-paid coerced sex, sexual harassment, rejecting use of condoms, slapping or pinching, verbal abuse, threats with a knife or some other objects, threatening that money and belongings would be taken, and rape or gang rape. The women perceived that violence was embedded within their working environment: “After the clients pay then we are treated as sexual objects to release their sexual pleasure; they have the right to beat us” [KI, aged 24 from beer bar]. Physical violence often occurred when clients were unsatisfied with the service and when the woman did not follow the client’s demands such as refusing to perform oral or anal sex: “He was very angry; he grabbed my hair while pulling my head to his cock and said ‘Do it for me now… suck it now!’” [22–30 years old FSW in FGD4].

Sex workers reported several strategies to avoid violence from customers, such as choosing clients carefully, and doing what clients asked for. One strategy was to carefully watch clients before deciding whether to have sex with them or not:

"“Clients prefer to spend hours talking and drinking before taking a girl for sex. While serving beers and snacks with some music, we observe and consider the clients and check references with friends” [KI, aged 24 from beer bar]."

Women reported that the risk of physical assault by clients was greater when clients tried to get cheap or free sex and additional time without paying. One woman illustrated how this risk could be reduced:

"“The agreement time had elapsed but he did not finish. Giving up the sex could have led to me being beaten. The best was to continue the sex; do what the client wants as quickly as possible and keep quiet till he is finished” [KI, aged 19 from beer bar & karaoke bar]."

The women also suggested that bringing a cell phone was a security measure when they went outside the working place with a client since it could be used if help was needed. Some stated that being able to visit a place where a security guard who can easily be asked for help during the service is available can also protect against violence.

Sex workers reported how they feared being arrested by police, especially on national holidays such as Lao National Day, during elections and on military days. Many complained that although all the official documents were completed and they had paid their working and village fees, the police still claimed the women were breaking the law. Strategies to reduce risk of violence/coercion included paying bribes, and being prepared to change location. Bribes were commonly reported:

4) Stigma and strategies to reduce stigma

"“I knew the ‘police’ wanted to have free services such as drinks, snacks and sex and my ‘pimp’ also encouraged me to do so. I managed to do this and the problem was solved. Some of them are our clients now, so we don’t have to worry next time” [18–31 years old FSW in FGD3]."

Women said that working in entertainment places increased the likelihood of being judged with disrespect due to perceived improper behavior. Fear of disclosure about one’s profession, e.g. being identified by relatives while working and meeting people who they knew, were great concerns. Feelings of shame and low self-value were frequently reported: “Sex workers are perceived as a source of diseases and cannot be accepted in this society” [KI, aged 24 from beer bar]. Another woman added: “I hate clients who call me *kalee* ‘bitch’, if they call me a bar woman it sounds better and I can feel happy with it. The word *kalee* hits my head suddenly” [18–24 years old FSW in FGD3].

Sex workers also worried about the impact of their work on love relationships. They feared being rejected and also being divorced if the profession was revealed after marriage. Such fears caused uncertainty and also made women leave love relationships when there was a risk of being disclosed. One woman described how her social image could stop her from marrying:

"“I am not really sure if we are going to marry. I am afraid that if my in-laws and husband find out about my work then they will say ‘how dare you step your foot into my house?’” [KI, aged 24 from beer bar & nightclub]."

One sex worker argued that there are many men who marry ‘a bitch’. In her opinion, some mind about this and some do not:

"“…if he married ‘a bitch’ and she turned to be good, people would praise her. Well, in the past, we may be bad, but now we turn to be good and build the family status” [17–26 years old FSW in FGD2]."

Sex workers also did not visit their families when sick in order to avoid stigma. They reported that people in the village stigmatized women returning to the village in poor health and often associated ill-health in women with having a deviant behaviour like selling sex. In order to avoid stigma and disgrace the sex workers avoided going home when sick.

"“I will not go home when ill because people would think that I do a bad job ‘sell sex’. I wait until I am better” [KI, aged 23 from beer bar & restaurant]."

On the other hand, some sex workers said that having money and wearing expensive clothes was also an effective method to reduce stigma. One woman said confidently that money could give her social status, despite her profession:

"“When I give up this work I will have money or things to show for my efforts. If not, then people could say ‘she did the work but she got nothing’. When I have money I can wear expensive clothes and use a good mobile phone. Then people will never look down on me” [18–31 years old FSW, FGD3]."

Another tactic recommended to avoid suspicious negative reactions was to wear inconspicuous clothing like *Sinn* ‘a Lao style skirt’, to not wear short dresses or use heavy cosmetics or coloring the hair when going home. Participation in village activities when going home was mentioned as an opportunity to get in contact with people they knew. Such contacts helped to show the women’s solidarity and respect of the traditional culture, which could significantly reduce negative perceptions.

5) Being cheated and preventive tactics to avoid being cheated

Another major source of risk cited by sex workers was being cheated. Those who cheated the women were fellow friends, clients, and boyfriends. Some women mentioned that friends had introduced them into their work without mentioning that they were expected to carry out sex work, as shown in the following quote:

"“At first I did not know that I had to have sex with clients because my friend told me that I just come to work as a hostess in the bar” [KI, aged 23 beer bar & restaurant]."

Sexual services usually took place outside the bars in places such as guesthouses, hotels, in the client’s room, or in a very remote area. Such unfamiliar environments increased the risk of being cheated and blackmailed by clients. Sex workers commonly reported that they were refused payment, or received lower payment than negotiated. Clients could also force sex workers to have sex for free by using a knife and or a sharp object as well as taking her money, mobile phone or other belongings. Strategies to avoid being robbed were to carry small sums of money and be clear in negotiations.

"“Don’t take lots of money. It can get you in trouble. Keep in mind that men who have sex with you are a type of brutal enemy” [18–31 years old FSW in FGD3]."

Other sex workers said that the most important thing to discuss was the type of service, the price and the length of the service before the agreement was made:

"“Before going out, I need to talk about what type the service is needed, short period or overnight stay, and that he must pay me first. If I do not get paid up-front, I will not go and have no reason to trust such unfamiliar clients” [KI, aged 31 from beer bar & restaurant]."

Being cheated by pimps was rarely reported. Some sex workers explained that pimps asking for money or payment for being in the bar were not cheating but provided a contribution to their business.

6) Social and economic insecurity and strategies to reduce this risk

The final source of risks mentioned by sex workers was social and economic insecurity. Women indicated that financial problems imposed mental and emotional distress because of less resources and ability to support their families. Causes of financial problems were health conditions (e.g. being infected with STIs and getting pregnant) and being cheated by clients, fellow friends or boyfriends. Women frequently reported not sharing information and ideas about health issues such as treatment of STIs, testing for HIV or pregnancy testing with their peers because of fears of being disclosed, leading to a lower income or even loss of work. A beer bar sex worker aged 30 from FGD5 said:

"“I don’t talk to friends about my genital symptoms because friends who are jealous of me might disclose it to clients. Also I may be asked to stop working for a while, or even not be allowed to work in this bar if the bar owner knows about this. I wait and quietly visit a health clinic alone”."

Many participants reported that they sometimes fought and competed amongst themselves for generous clients. This created mistrust between those working at the same venue:

"“I don’t trust anyone. No one is worth trusting in this venue and they are all in business” [KI, aged 18 from nightclub]."

Even romantic relationships were avoided because of the risk of economic loss. It was mentioned in all FGDs that engaging in sex work is for money but not for love. Having a love relationship takes time away from making money:

"“Having a boyfriend is a weak point. The guy would give us some money first then he would find reasons to get money from us and because of love we give him the money that we earned” [18–24 years old FSW in FGD1]."

### Perceived benefits and strategies to increase benefits

In addition to telling us about the risks of sex work, participants spontaneously reported three categories of benefits related to their work: 1) financial security and ways to increase income, 2) fulfilling social obligations and getting self-value and 3) sexual pleasure and strategies to increase pleasure (Table 2). These benefits and strategies to increase benefits are illustrated below.

1) Financial security and ways to increase income

**Table 2:** Positives outcomes of sex work in Laos and strategies to increase chances to achieve them

**Strategies**	**Benefits**
Easy and quick money	Financial security
Accept sex without a condom	
Accept oral and anal sex	
Offer a non-alternative sex	
Pay off debts	Fulfilling social obligations
Self sufficient	
Support family	
Secure for future	
Select handsome clients	Sexual pleasure
Don’t use condom if client looked clean	
Serve many clients	
Spent time with clients	

Participants reported that sex work is an easy and good source of income compared to other jobs. They said that sex work was suitable for a low-educated person because working in a bar does not require formal training or skills and is quickly learned.

"“When I first started working in this bar I did not know how to take care of clients. I spent time observing and asked my friend who worked in the same bar. Soon I could work myself” [18–24 years old FSW in FGD1]."

The sex workers mentioned strategies for increasing their income e.g. engaging in risky sex and agreeing on the client’s sexual requests.

"“When we arrived at the hotel room, he did not want to use a condom based on the agreement. I asked him to give me more money… and then I got three times higher than the negotiated price” [KI, aged 24 from beer bar]."

"“Many clients need oral sex before vaginal or anal sex or both and you can use that to ask for more pay. Usually, men choose pleasure, but not the price” [18–30 years old FSW in FGD5]."

Sex workers explained that men want to visit sex workers because sex at home is not pleasurable and being nice to clients will give more benefits. One young woman applies this concept at first encounter with clients in the following way:

2) Fulfilling social obligations and getting self-value

"“When clients visit, I said Sabaidee ‘hello sir’ while smiling. I immediately invite them for a seat. I serve a glass of cold water first, talk to them softly and show my respect throughout the service. It doesn’t matter if I go for sex but in the end I usually have a very good tip” [KI, aged 19 from beer bar & karaoke bar]."

Another benefit from sex work that participants reported was fulfilling social obligations and getting self-value. Most women said that they engaged in sex work to support their families and care for the parents. They regularly sent money to their families and the amount depended on how much they could earn. Due to the poor living conditions and lack of employment opportunities in their home villages, some women said that they followed friends who worked as bar workers in the “big city” to find alternative ways to make a living and support their families.

"“After the wet rice farming season there is nothing much to do at home. My parents are farmers and they cannot raise many of us. Taking this job helps contribute to my family’s income” [KI, aged 18 from nightclub]."

Some women had dropped out of school because they needed to find a job in order to support their family. This motivated many of them to engage in sex work.

"“My parents borrow money to hire people for planting rice but the rice was dead after the flooding. They had to pay for the capital ‘the rice seed and the labor.’ If they could not pay back, our land would be confiscated. I don’t go to school and must work in the bar to gradually pay back the debt” [KI, aged 24 beer bar & nightclub]."

Another participant explained her situation in the following way:

"“In my case, I have to take responsibility for all of my parents’ expenses, even my brothers’ and sisters’ school’s fees. I decided do the job myself because there are many people who need my support” [24–31 years old FSW in FGD3]."

Some find the work very hard but a necessary fact of life as illustrated in the following citation:

"“I had first sex with a guy over here, after working in the bar. I got the ‘sell the virginity’ [deflowering fee] and that was the first money I earned. I saved some money for myself and also sent some to my parents” [KI, aged 26 from beer bar]."

Being able to support the family made participants proud and gave them potential future social worth, despite the stigma they experienced in the present.

"“I think that whatever they say to insult us, one day in the future they will praise us if we do good things for our family” [KI, aged 31 from beer bar and restaurant]."

Many women said that being a daughter requires obedience and sacrifice to the family’s demands, and that they were proud to be able to support the family.

3) Sexual pleasure and strategies to increase sexual feelings

"“Parents raise us since we were born, they dedicated a lot already. When they were sick it was hard for them to pay for health care. As a daughter, we cannot dare to see such them suffer. It is time to work for them now” [22–31 years old FSW in FGD3]."

A third benefit of sex work reported by many participants was sexual pleasure. Some sex workers claimed that having sex with clients did not give them pleasure because they only did it for money but without love: “Sexual pleasure with clients… I did not have such feeling because I did not love them and without love then not possible to have it” [KI, aged 18 from nightclub].

Another beer bar woman added:

"“When the clients finish then I get my money. They usually finish quick, but for me it takes time. But sometimes it is really great” [17–22 years old FSW in FGD4]."

Many sex workers noted that spending time to get sexual pleasure was lost time and they were reluctant to reach ‘sexual climax’ since they needed to find more clients, as this young women noted:

"“If I reached the peak point ‘orgasm’ then I will be very tired and then I cannot find more clients” [KI, aged 24 from beer bar]."

In contrast, many other women argued that sex work gave them not only money but also sexual pleasure and fun:

"“Working in the bar I have money, and I also enjoy sex” [24–31 years old FSW in FGD3]."

One beer bar worker remarked that she preferred to have polite and handsome clients and that such clients could increase her sexual pleasure:

"“Most of the time I select clients not only because of money but because they are both good looking and have good money. I like a good looking and clean one, it makes really great when having sex” [KI, aged 18 from nightclub]."

## Discussion

The results from the present study indicate that the most common risks experienced by the interviewed sex workers were STIs/HIV, unintended pregnancy, stigma, violence, being cheated, and social and financial insecurity. Similar findings have been reported from other settings [3,19]. Condom breakage or clients removing the condom during the transaction were the greatest risk for STIs/HIV infections and unintended pregnancy but these problems were difficult to avoid. However, even when possible, the women did not always avoid such risks if they also perceived “benefits” in the situation. For example, sex without a condom, and oral or anal sex could result in more money. Existing health behaviour research has shown that “risks and benefits” are the most powerful predictors of HIV related behaviour change [20,21]. In other words, sex workers may adopt “risky behaviour” when the perceived benefits from non-condom use (e.g. financial gain) are perceived as greater than the benefit of using condoms (e.g. protection against STIs) [22]. A study from Vietnam noted that when clients refused to use a condom, FSWs charged more money and went ahead having sex without a condom [23].

Based on sex workers’ accounts, violence and unintended pregnancy were also great concerns in their working environment. Clients’ violence threatened sex workers’ well-being and sometimes resulted in unintended pregnancy. However, participants perceived these risks as part of their working environment. A lesson learned from Thailand suggested that sex workers experienced violence as a result of social norms, the legal system, and politics, but they were prepared to accept violence as a condition of the working environment [19].

Violence from clients not only contributed to risk of getting pregnant but also resulted in emotional distress in this study. In many cultures, including Lao, pregnancy outside of marriage is strongly condemned and viewed as a source of social stigma bringing shame to the women and her family [24], often resulting in unsafe abortion [25]. In a survey among 1,421 FSWs from Laos, 51 percent were forced by a client not to use a condom and 89 percent had been pregnant in the past 6 months. Most of these pregnancies resulted in an abortion [1]. Our findings suggest a high degree of vulnerability of sex workers in this particular setting. Sex workers’ own strategies for avoiding unwanted pregnancy as a result of violence as identified in this study e.g. use of ‘dual protection’ should be promoted and facilitated.

The results of this study indicate that sex workers in Laos are not only victims but they also have control over their working situation to some extent and that they see benefits regarding their work. Similar findings have been reported from Pakistan [26]. In our study, sex workers saw financial stability as a benefit of sex work, allowing them to support their families, and thus fulfill important social obligations. However, in some cases this “control” seems to be superficial, such as when financial benefits outweigh the risks of STIs, unwanted pregnancy, and stigma. As mentioned above, our study participants described that they sometimes decided to follow the client’s needs e.g. having sex without a condom, or having oral or anal sex, but that this was in order to get better pay. This behaviour indicates that sex workers make decisions to advance their interests. Central to the Health Belief Model is the concept that knowledge leads to heightened perceived risks, which leads to behaviour change [27]. On the other hand, several studies have found no relationship between perceived risk of HIV infection and the adoption of health protective behaviours or intention to change behaviour [20,21,28]. The role of perceived benefits may be stronger than perceived risks because of the wider social realities in which sex workers live. In a study in the same setting, women said that they engaged in sex work because of poverty and the need to provide financial support to their family (Phrasisombath K, Thomsen S, Sychareun V, Faxelid E: ‘Health is Wealth and Wealth is health’-perception of health and ill-health among female sex workers in Savannakhet province, Laos, 2011. (Under review, Global Health Action, 2012)). Thus, sex workers in Laos may not be completely empowered to apply their knowledge because of structural factors such as poverty and social obligations.

Sex workers’ lives are not only framed by poverty, but also by social and cultural norms such as gender roles. Economic and gender inequities together increase the risk of HIV infection, especially among women [29,30]. In many contexts, including Lao, when families are stressed financially, girls are often kept out of school [31]. Thus women do not qualify for well-paid jobs, have fewer job opportunities, and are financially vulnerable, forcing some women to engage in commercial sex as a mean of survival [26]. In contexts where there is unequal distribution of wealth and resources, obligations to support one’s family are of utmost importance for the survival of the family. This obligation might, however, lead to high-risk sexual behaviour. For example, when sex workers need to support their families or pay the family’s debt they may force themselves to work harder and accept unsafe sex as shown in our study. Being able to support the family made sex workers proud and gave them social value. Although selling sex is perceived negatively and stigmatized and not accepted by the society, the women hoped for a possibility to be accepted when they had fulfilled the family’s needs and social expectations, as have been reported also from Vietnam [32,33].

What is interesting in this study is that participants had appeared to exert some control over the prevailing gender norms. They were taking their economic livelihoods into their own hands in order to fulfill social obligations towards their families. The women in our study even sometimes choose to sacrifice romantic relationships in order to safeguard their income. In this sense, the sex workers in our study were in control of their lives. In other ways, sex workers in this context were disadvantaged and had limited power over their own lives since they were dependent on the goodwill of the police and other authorities.

**Figure 1:**
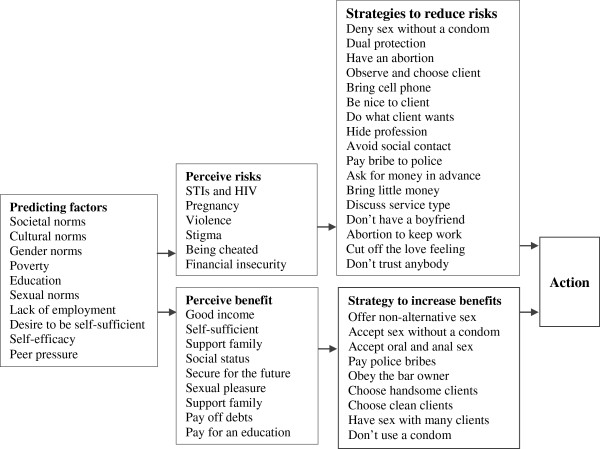
**Potential pathway of risks, benefits and survival strategies that linked to sexual practices among FSWs in Laos.** Adapted from Carmel S, 1990; the Health Belief Model in the research of AIDS-related preventive behavior.

In order to illustrate the findings of this study in relation to the Health Belief Model we have created a diagram of the risks, benefits and strategies that sex workers in Laos use when choosing action (Figure 1). This figure shows that risks and benefits are sometimes contradictory to each other, forcing sex workers in this particular study context to make decisions that they know are harmful, but which they feel they cannot avoid due to social, economic and cultural factors. This point to the importance of adapting theories such as the Health Belief Model to different contexts and environments [28]. Sex workers are often driven by financial need to behave in risky manners, even though they are aware of the risks. In Laos, female sex workers seem to have another layer of demand on them that affect their decision-making even more – the responsibility to their families. Understanding these pressures and the strategies that sex workers in Laos use to negotiate them will allow us to find strategies to help them in these difficult choices.

### Methodological considerations

Applying group discussions and in-depth interviews allowed participants to discuss their perceptions, providing insight into their working experiences and concerns. Furthermore, involving researchers from different backgrounds such as social science, midwifery and medicine helped to check consistency and validate the data. In addition, the private venue for the group discussions and interviews created an environment where the participants could freely discuss personal issues. Discussing sex-related matters is sensitive and participants might be shy about speaking out. Therefore, we involved peer educators who used to work as FSWs to enhance the trustworthiness of the findings. However, a limitation could be that the women might have felt embarrassed talking about these issues in front of a male moderator/interviewer (the first author). In order to minimize such barriers one FGD and one individual interview using male moderator/interviewer was pilot tested prior to the data collection. On the other hand, using a male moderator/interviewer might have made it difficult to obtain sensitive, personal experiences such as sexual issues from the participants. Furthermore, the adapted Health Belief Model used in our study did not cover other elements such us “Susceptibility” and “Cues for action”. Despite these limitations, the study provided valuable information regarding FSW’s perceptions about risk, benefit and working environment in Laos. We believe that what the participants shared is also valid for other FSWs in other similar settings.

## Conclusions

FSWs decision-making and risk taking behaviour were outcomes of risk-benefit analysis and were fueled by gender inequities and cultural and social norms that reinforce women’s lower social and economic status. Health education on STI/HIV prevention should be promoted and sex workers should be supported in their desires to be dually protected from both STIs/HIV and unwanted pregnancy. Bar owners or pimps should be trained and involved in order to provide health information, advocate FSWs to empower them to perform safe sex. Finally, addressing women’s poverty, and gender-power inequities may be more effective in reducing HIV and other risks related to sex work in these particular settings.

## Competing interests

The authors have declared that no competing interests exist.

## Authors’ contributions

KP is the main author; he developed the research design, prepared data collection, supervised research assistants during data collection, carried out the analysis, and drafted the manuscript. ST and EF assisted with the research design, collaborated in the analysis and offered critical comments in reviewing the manuscript. VS assisted with the research design, provided logistic supports and cooperate during field-work. All authors read and approved the final manuscript.

## Sources of support

Health Systems Research Program (HSRP), Lao PDR and Sida, Sweden.

## Pre-publication history

The pre-publication history for this paper can be accessed here:

http://www.biomedcentral.com/1471-2458/12/1004/prepub
